# Brain Work and Over-Work

**Published:** 1880-04

**Authors:** 


*Brain Work and Overwork.* By Dr. H. C. Wood, Clinical Professor of Nervous Diseases University of Pennsylvania, Etc. Publisher: Presley Blackiston, 1012 Walnut Street, Philadelphia, 1880.

This manual is another of the series known as "American Health Primers," which have passed into the hands of the new Publisher above named, and, like its predecessors, contains much that is instructive and interesting. The introduction relates to the supposed increase of nervous affections, the author contending that the popular belief in such an increase rests only upon the superiority of modern diagnosis, we recognizing them more clearly than did our fathers. The general causes of nervous trouble are then discussed, such as exposure, sexual excesses, alcohol, tea and coffee, and gluttony. The effects of emotional and intellectual work, difference in labor power of sexes, and woman's work, proper time of work, variety of work, rest in recreation, and rest in sleep, are some of the many divisions of the subject skillfully treated by the author. Concluding with "signs of nervous breakdown."

The publishers of this series of "Health Primers" have been very successful in introducing such subjects as interest all classes, as the contents show, and also in securing authors competent for the work.

